# *PPARD* rs2016520 (T/C) and *NOS1AP* rs12742393 (A/C) polymorphisms affect therapeutic efficacy of nateglinide in Chinese patients with type 2 diabetes mellitus

**DOI:** 10.1186/s12920-021-01108-5

**Published:** 2021-11-12

**Authors:** Tao Wang, Jin-Fang Song, Xue-Yan Zhou, Cheng-Lin Li, Xiao-Xing Yin, Qian Lu

**Affiliations:** 1 Jiangsu Key Laboratory of New Drug Research and Clinical Pharmacy, Xuzhou Medical University, Xuzhou, China; 2 Department of Pharmacy, Affiliated Hospital of Xuzhou Medical University, Xuzhou, China; 3 Department of Pharmacy, Affiliated Hospital of Jiangnan University, Wuxi, China

**Keywords:** *PPARD*, *NOS1AP*, Genetic polymorphism, Type 2 diabetes mellitus, Nateglinide

## Abstract

**Background:**

Genetic polymorphisms in the *PPARD* and *NOS1AP* is associated with type 2 diabetes mellitus (T2DM); however, there is no evidence about its impact on the therapeutic efficacy of nateglinide. This study was designed to investigate a potential association of *PPARD* rs2016520 (T/C) and *NOS1AP* rs12742393 (A/C) polymorphisms with efficacy of nateglinide in newly diagnosed Chinese patients with type 2 diabetes mellitus (T2DM).

**Methods:**

Sixty patients with newly diagnosed T2DM were enrolled to identify *PPARD* rs2016520 and *NOS1AP* rs12742393 genotypes using the polymerase chain reaction-restriction fragment length polymorphism assay (PCR–RFLP). All subjects were treated with nateglinide (360 mg/day) for 8 weeks. Anthropometric measurements, clinical laboratory tests were obtained at baseline and after 8 weeks of nateglinide treatment.

**Results:**

After nateglinide treatment for 8 consecutive weeks, patients with at least one C allele of *PPARD* rs2016520 showed a smaller decrease in post plasma glucose (PPG), homeostasis model assessment for beta cell function (HOMA-B) than those with the TT genotype did (*P* < 0.05). In patients with the AA genotype of *NOS1AP* rs12742393, the drug showed better efficacy with respect to levels of fasting plasma glucose (FPG), fasting serum insulin (FINS), HOMA-B and homeostasis model assessment for insulin resistance (HOMA-IR) than in patients with the AC + CC genotype (*P* < 0.05). *NOS1AP* rs12742393 genotype distribution and allele frequency were associated with responsiveness of nateglinide treatment (*P* < 0.05).

**Conclusions:**

The *PPARD* rs2016520 and *NOS1AP* rs12742393 polymorphisms were associated with nateglinide monotherapy efficacy in Chinese patients with newly diagnosed T2DM.

**Trial registration:**

Chinese Clinical Trial Register ChiCTR13003536, date of registration: May 14, 2013.

## Introduction

Nateglinide is an important non-sulfonylurea oral hypoglycemic agent that promotes insulin secretion from pancreatic islet beta cells by inhibiting ATP-sensitive K^+^ channels and activating Ca^2+^ channels [1, 2]. However, considerable interindividual differences in the therapeutic efficacy of nateglinide have been reported in patients with T2DM [1, 2]. The underlying mechanism is still unclear. It is hypothesized that genetic polymorphisms of genes that code drug metabolizing enzymes, drug transporters, drug targets, or susceptibility genes related to T2DM pathogenesis may affect the pharmacokinetic or pharmacodynamics process of drugs, and eventually lead to interindividual variation in therapeutic efficacy of nateglinide [30]. Cytochrome P450 (CYP) 2C9 and CYP3A4 have been identified as the main metabolic enzymes involved in the biotransformation of nateglinide [3]. SLCO1B1 gene encoding organic anion transporting polypeptide 1B1 (OATP1B1), which is involved in cellular uptake and transport of nateglinide [3]. The interindividual differences may be attributed to genetic polymorphism of *CYP2C9* and *SLCO1B1*, but not the *CYP3A4* polymorphisms [3]. Nevertheless, this could not elucidate all the causes of various nateglinide responses [4, 5].

*PPARD* is located on chromosome 6p21.1-p21.2, and its coding product PPAR-δ (also named PPAR-β) is a member of the peroxisome proliferator activated receptor family, which is widely distributed in the liver, kidneys, cardiac and skeletal muscle, adipose tissue, brain, pancreatic and vasculature [6]. PPAR-δ plays an important role in insulin resistance and islet β-cell function [7–9]. More recently, PPAR-δ activation came into focus as an interesting novel approach for the treatment of metabolic syndrome. Meanwhile, both preclinical and clinical studies have shown that PPAR-δ specific agonist therapy enhanced β-oxidation, decreased free fatty acid, and improved insulin sensitivity [10, 11]. Large-scale clinical studies in the Chinese population have shown that *PPARD* rs2016520 polymorphism (also named + 294 T > C or − 87 T > C) is associated with blood glucose, insulin level and insulin resistance, and is a key factor affecting the development of metabolic syndrome and T2DM [12, 13]. Studies in a Mexican population have produced similar results [14].

*NOS1AP*, located on chromosome 1q22.3, and its coding product is known as carboxy-terminal PDZ ligand of neuronal nitric oxide synthase (nNOS), which regulates nNOS activity through interaction with the PDZ binding region of nNOS [15]. It was shown that elevated levels of Ca^2+^ in islet β-cells can activate nNOS in the surface layer of insulin secretory granules and participate in the insulin secretion process [15]. In addition, the administration of cholesterol to inhibit nNOS activity can impede the process of insulin secretion, further suggesting its biological role in insulin secretion [16, 17]. The *NOS1AP* rs12742393 polymorphism was found to be associated with susceptibility to new-onset diabetes in patients treated with calcium channel blockers in different populations, and pharmacogenomic studies have shown that *NOS1AP* polymorphisms are one of the important factors contributing to differences in the efficacy of hypoglycemic agents, especially sulfonylureas [18, 19]. One clinical study showed that *NOS1AP* rs12742393 C allele gene was associated with an increased susceptibility to T2DM in the Chinese population [20]. Though the studies on how the variants influenced the diseases were limited, one functional study showed that rs12742393 could affect *NOS1AP* expression through influencing transcription factor binding [21]. To date, there has been no report about the effect of *PPARD* rs2016520 and *NOS1AP* rs12742393 polymorphism on nateglinide response. Therefore, the *PPARD* rs2016520 and *NOS1AP* rs12742393became our focus.

Based on the facts that *PPARD* and *NOS1AP* play crucial roles in functional regulation of β-cells, insulin resistance and metabolism, we conduct this study to identify the association *PPARD* rs2016520 (T/C) and *NOS1AP* rs12742393 (A/C) polymorphisms with therapeutic efficacy of nateglinide in Chinese patients with T2DM.

## Materials and methods

### Study design and participants

A total of 78 T2DM patients were recruited for this study according to the inclusion and exclusion criteria, of which 60 (39 men and 21 women) completed the full follow-up and study process. Inclusion and exclusion criteria were developed based on previous studies and T2DM was diagnosed according to the 1999 World Health Organization criteria [18, 22]. Inclusion criteria were (i) BMI in the range of 18.5–30 kg/m^2^ and (ii) patients who were newly diagnosed with T2DM, unmedicated and had never use agonists or inhibitors of CYP2C9, CYP3A4 and OATP1B1. Patients on insulin therapy, pregnant or lactating women, and patients with serious diseases such as acute myocardial infarction, cerebrovascular accident, trauma, and severe hepatic and renal disorders were excluded. All subjects who met the enrollment criteria received 360 mg nateglinide per day (120 mg once before meal) orally for 8 consecutive weeks. The study was registered in the Chinese Clinical Trial Register (No. ChiCTRCCC13003536), in which the protocol used was approved by the ethics committee of the Affiliated Hospital of Xuzhou Medical University. All methods were carried out in accordance with Ethical Review of Drug Clinical Trials in China and followed the Helsinki Declaration II. Written informed consent was obtained from each participant before the study.

### Anthropometric and biochemical measurements

The general anthropometric parameters considered for this study were height (in meters), weight (in kilograms), and waist and hip circumferences (in centimeters). After an overnight fast by the study subjects, blood samples for measurements of plasma glucose and insulin were obtained both in the fasting state and 2 h later during a standard 75-g oral glucose tolerance test. Plasma glucose and insulin, hemoglobin A1c (HbA1c), and serum lipids were measured as previously described [22]. These parameters were measured at the end of weeks 0 and 8 after administration of nateglinide. The homeostasis model assessment for insulin resistance (HOMA-IR) and beta cell function (HOMA-B) are given by: HOMA-IR = fasting insulin level (mU/L) × fasting glucose level (mmol/L)/22.5; HOMA-B = 20 × fasting serum insulin (FINS)/(FPG-3.5) [23].

### Genotyping

Genomic DNA was extracted from peripheral blood leucocytes using a SiMax Genome DNA Kit (Sbsbio, Shanghai, China). In the present study, the *PPARD* rs2016520 locus was amplified by the polymerase chain reaction (PCR) with the following primers: 5-TGGGAAGGGTGATAGGGCA-3 (forward) and 5'-CTGGTGAGTGGCAGAGCAGA-3 (reverse). The 602 bp PCR products were digested by FoKI (NEB, Beijing, China). For the *NOS1AP* rs12742393 locus, the following primer pairs were used 5-GGTGAATGTGTACAAAGGAGAAGG-3 (forward) and 5-CAAACTGAAAT GGACCACAAAGAG-3 (reverse). The PCR products were digested by BsrI (NEB, Beijing, China). All obtained DNA fragments were separated by 2% agarose gel electrophoresis followed by ethidium bromide staining and visualization with UV transillumination. To confirm the assay results, 5.0% of all samples were directly sequenced.

### Definition of the response to nateglinide

T2DM subjects were classified into two groups based on changes in HbA1c after treatment with nateglinide: responder and non-responder. According to previous studies, nateglinide monotherapy improved HbA1c in patients with T2DM by an average of 10% to 20% from baseline levels [24–26]. In the present study, HbA1c levels were reduced by an average of 19.95% in all subjects treated with nateglinide. Therefore, we identified a 20% improvement in HbA1c after 8 weeks of nateglinide treatment as an intermediate value, with responders were defined as patients with 20% or greater decrease in HbA1c and non-responders defined as patients who failed to achieve this level.

### Statistical analysis

Statistical analyses were carried out using SPSS software (version 16.0, SPSS Inc., Chicago, IL). The measurement data and count data were expressed as mean ± standard deviation (SD) and proportion, respectively. Frequencies of genotypes were assessed using χ^2^ tests in the study sample. Parameters before and after treatment were compared by paired *t*-test. Independent samples *t* tests were used to estimate the effects of nateglinide on biochemical index among genotypes [22]. Parameters with nonnormal distribution were analyzed by the Kruskal–Wallis test. α = 0.05 was used as the test level.

## Results

### Clinical efficacy evaluation of nateglinide

To evaluate the effects of *PPARD* and *NOS1AP* variations on the efficacy of nateglinide, 60 newly diagnosed T2DM patients with various *PPARD* rs2016520 (C/T) and *NOS1AP* rs12742393 (A/C) genotypes but with the same *SLCO1B1* T521C and CYP2C9*1 genotype were enrolled. Nateglinide significantly decreased the levels of FPG, PPG, HbA1c, TG, and TC, and increased the levels of FINS, PINS, HOMA-B and HDL-C levels in patients with T2DM after 8 weeks of nateglinide treatment (Table 1).

**Table 1:** Clinical characteristics of T2DM patients before and after nateglinide treatment

Parameters	Before treatment	After treatment	*P* values
FPG (mmol/L)	8.70 ± 2.23	6.67 ± 1.17	0.000
PPG (mmol/L)	14.26 ± 2.94	10.46 ± 1.59	0.000
FINS (mU/L)	9.26 ± 5.87	14.09 ± 13.47	0.007
PINS (mU/L)	39.33 ± 33.11	70.21 ± 52.89	0.000
HOMA-IR	3.61 ± 2.42	4.27 ± 4.28	0.246
HOMA-B	28.26 ± 16.01	60.31 ± 35.32	0.000
HbA1c (%)	8.35 ± 1.74	6.71 ± 1.00	0.000
TG (mmol/L)	2.15 ± 1.34	1.85 ± 1.10	0.002
TC (mmol/L)	4.98 ± 1.31	4.58 ± 1.08	0.001
HDL-C (mmol/L)	1.30 ± 0.42	1.49 ± 0.68	0.026
LDL-C (mmol/L)	2.82 ± 0.78	2.68 ± 0.74	0.066

Abbreviations: FPG, fasting plasma glucose; PPG, postprandial plasma glucose; FINS, fasting serum insulin; PINS, postprandial serum insulin; HOMA-IR, homeostasis model assessment for insulin resistance; HOMA-B, homeostasis model assessment for beta cell function; HbA1c, hemoglobin A_1c_; TG, triglyceride; TC, total cholesterol; HDL-C, high-density lipoprotein-cholesterol; LDL-C, low-density lipoprotein-cholesterol

Data are expressed as mean ± SD. *P* values are determined by the Student’s *t* test

### Effects of the rs2016520 and rs12742393 polymorphisms on therapeutic efficacy of nateglinide in patients with T2DM

**Fig. 1:**
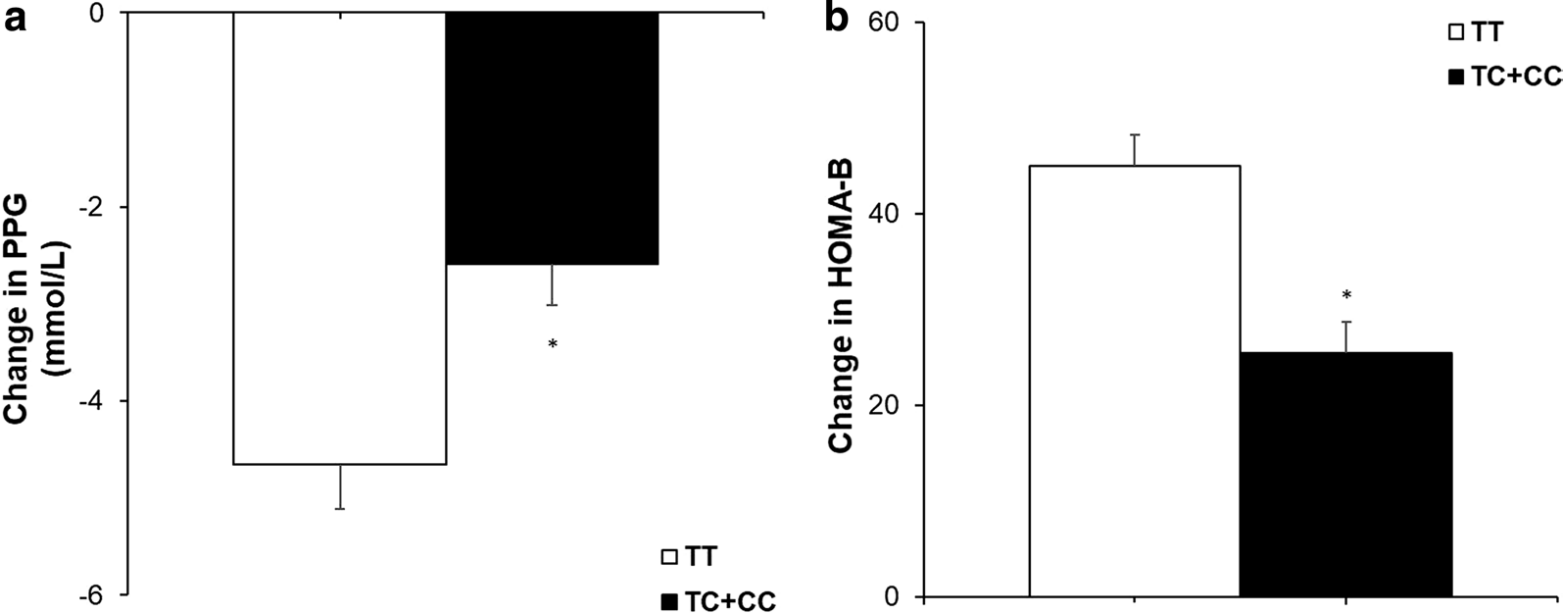
Comparisons of DV (postadministration minus preadministration) of PPG (**a**) and HOMA-B (**b**) between the different PPARD rs2016520 genotypes in T2DM patients after treatment of nateglinide. Data are expressed with mean ± standard error. *P < 0.05 compared with TT genotype group

**Fig. 2:**
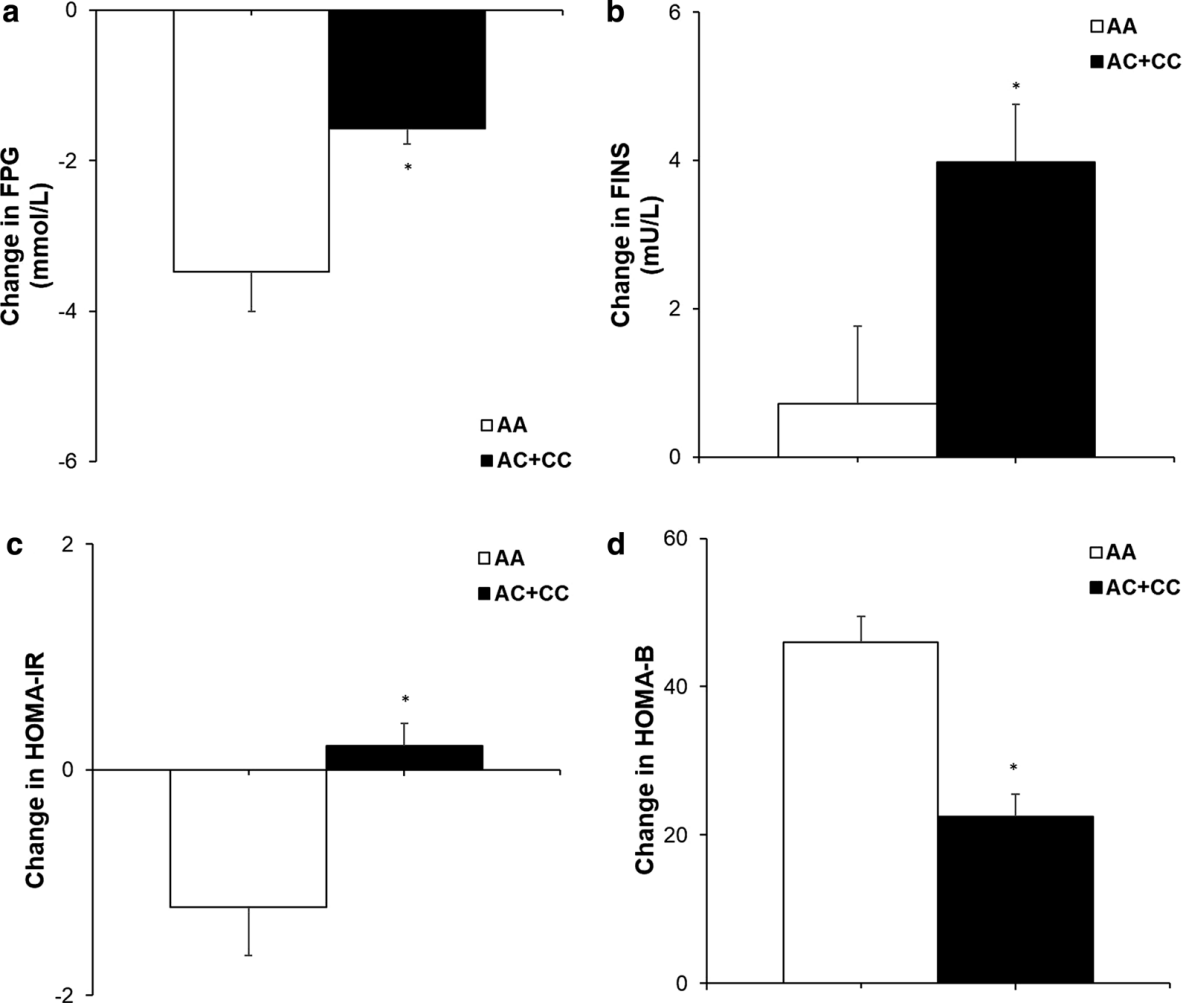
Comparisons of DV (postadministration minus preadministration) of FPG (**a**), FINS (**b**), HOMA-IR (**c**) and HOMA-B (**d**) among the different NOS1AP rs12742393 genotypes in T2DM patients after treatment of nateglinide. Data are expressed with mean ± standard error. *P < 0.05 compared with AA genotype group

Patients with *PPARD* rs2016520 TT genotypes had a significantly decrease in PPG and notably increase HOMA-B as compared with patients with the TC + CC genotypes, which indicated that patients with genotype TT had better efficacy of nateglinide monotherapy (Table 2, Fig. 1). Moreover, patients with *NOS1AP* rs12742393 AC + CC genotypes had poor response of nateglinide with respect to FPG, FINS, HOMA-IR, and HOMA-B compared with AA genotype carriers (Table 3, Fig. 2).

**Table 2:** Effects of different *PPARD* rs2016520 genotypes in T2DM patients on clinical characteristics determined before and after nateglinide treatment

Parameters	*PPARD* rs2016520			*P* values
		TT	TC + CC	
N (male/femal)		35(24/11)	25(15/10)	0.493^b^
FPG (mmol/L)	Before	9.07 ± 2.63	8.19 ± 1.42	0.134
	After	6.74 ± 1.36	6.57 ± 0.87222	0.579
	DV	− 2.32 ± 1.72	− 1.61 ± 1.44	0.099
PPG (mmol/L)	Before	15.23 ± 2.78	12.90 ± 2.66	0.002
	After	10.57 ± 1.71	10.31 ± 1.42	0.538
	DV	− 4.66 ± 2.70	− 2.59 ± 2.13	0.002
FINS (mU/L)	Before	9.36 ± 5.93	9.13 ± 5.91	0.883
	After	16.06 ± 15.72	11.32 ± 9.05	0.182
	DV	6.70 ± 15.03	2.19 ± 10.33	0.201
PINS (mU/L)	Before	41.55 ± 30.72	36.23 ± 36.61	0.544
	After	75.14 ± 54.71	63.29 ± 50.51	0.397
	DV	33.59 ± 43.71	27.07 ± 29.25	0.519
HOMA-IR	Before	3.81 ± 2.65	2.72 ± 1.47	0.443
	After	4.94 ± 4.94	3.34 ± 2.97	0.157
	DV	1.12 ± 4.85	− 0.01 ± 3.53	0.338
HOMA-B	Before	28.25 ± 16.60	25.22 ± 14.80	0.469
	After	73.20 ± 42.81	50.74 ± 26.43	0.015
	DV	44.95 ± 19.23	25.48 ± 15.93	0.000
HbA1c (%)	Before	8.58 ± 1.81	7.02 ± 1.60	0.221
	After	6.58 ± 1.12	6.42 ± 0.80	0.543
	DV	− 1.87 ± 1.55	− 1.60 ± 1.44	0.714
TG (mmol/L)	Before	2.19 ± 1.58	2.09 ± 0.92	0.782
	After	1.90 ± 1.26	1.78 ± 0.83	0.690
	DV	− 0.29 ± 0.78	− 0.31 ± 0.64	0.926
TC (mmol/L)	Before	4.67 ± 1.20	4.45 ± 0.88	0.906
	After	4.74 ± 1.19	4.52 ± 0.87	0.439
	DV	− 0.29 ± 0.75	− 0.55 ± 1.12	0.283
HDL-C (mmol/L)	Before	1.29 ± 0.45	1.31 ± 0.38	0.788
	After	1.38 ± 0.41	1.64 ± 0.92	0.158
	DV	0.09 ± 0.38	0.32 ± 0.87	0.183
LDL-C (mmol/L)	Before	2.79 ± 0.80	2.87 ± 0.78	0.720
	After	2.75 ± 0.79	2.58 ± 0.67	0.364
	DV	− 0.04 ± 0.61	0.29 ± 0.57	0.110

Abbreviations: FPG, fasting plasma glucose; PPG, postprandial plasma glucose; FINS, fasting serum insulin; PINS, postprandial serum insulin; HOMA-IR, homeostasis model assessment for insulin resistance; HOMA-B, homeostasis model assessment for beta cell function; HbA1c, hemoglobin A_1c_; TG, triglyceride; TC, total cholesterol; HDL-C, high-density lipoprotein-cholesterol; LDL-C, low-density lipoprotein-cholesterol

Data are given as mean ± SD. *P* values represent statistical difference between different genotypes assessed by independent-samples t-tests. ^b^*P* values are determined by the Pearson chi-square test. ^c^*P* values are determined by the Kruskal–Wallis test

DV, differential values (post-administration minus pre-administration)

**Table 3:** Comparisons of clinical characteristics in T2DM patients with different *NOS1AP* rs12742393 genotypes before and after nateglinide treatment

Parameters		AA	AC + CC	*P* value
N(male/female)		24(17/7)	36(22/14)	0.598^b^
FPG (mmol/L)	Before	9.97 ± 2.54	10.32 ± 2.02	0.580
	After	6.51 ± 1.34	8.75 ± 1.44	0.000
	DV	− 3.48 ± 2.55	− 1.57 ± 1.28	0.000^c^
PPG (mmol/L)	Before	17.25 ± 4.31	16.73 ± 4.50	0.651
	After	10.47 ± 3.45	12.95 ± 3.68	0.016
	DV	− 6.78 ± 4.41	− 4.81 ± 3.57	0.000^c^
FINS (mU/L)	Before	9.33 ± 6.49	8.92 ± 5.96	0.802
	After	10.04 ± 6.26	12.87 ± 6.81	0.110
	DV	0.72 ± 5.15	3.98 ± 4.65	0.014
PINS (mU/L)	Before	31.63 ± 22.32	31.90 ± 21.62	0.963
	After	46.91 ± 26.82	47.82 ± 26.90	0.028
	DV	14.31 ± 14.23	17.36 ± 15.33	0.441
HOMA-IR	Before	4.04 ± 2.96	4.02 ± 2.58	0.978
	After	2.81 ± 1.66	4.32 ± 2.21	0.006
	DV	− 1.22 ± 2.07	0.21 ± 1.23	0.001
HOMA-B	Before	25.45 ± 17.21	27.01 ± 16.92	0.730
	After	75.20 ± 43.81	47.7 ± 39.31	0.014
	DV	45.95 ± 37.23	22.48 ± 21.93	0.003
HbA1c (%)	Before	9.81 ± 1.89	9.68 ± 1.96	0.809
	After	7.02 ± 0.78	7.01 ± 1.74	0.979
	DV	− 2.79 ± 1.58	− 2.71 ± 1.28	0.830
TG (mmol/L)	Before	2.21 ± 1.53	2.51 ± 2.26	0.572
	After	1.84 ± 1.04	2.06 ± 2.02	0.625
	DV	− 0.36 ± 1.13	− 0.37 ± 2.04	0.983
TC (mmol/L)	Before	5.10 ± 1.01	5.32 ± 1.78	0.585
	After	5.04 ± 0.91	4.74 ± 1.23	0.311
	DV	− 0.06 ± 0.91	− 0.54 ± 1.47	0.806
HDL-C (mmol/L)	Before	1.41 ± 0.42	1.39 ± 0.49	0.871
	After	1.37 ± 0.39	1.30 ± 0.43	0.524
	DV	− 0.06 ± 0.45	− 0.11 ± 0.66	0.747
LDL-C (mmol/L)	Before	3.10 ± 0.82	3.19 ± 1.16	0.744
	After	3.31 ± 0.91	3.02 ± 1.12	0.295
	DV	0.21 ± 0.92	− 0.03 ± 1.37	0.455

Abbreviations: FPG, fasting plasma glucose; PPG, postprandial plasma glucose; FINS, fasting serum insulin; PINS, postprandial serum insulin; HOMA-IR, homeostasis model assessment for insulin resistance; HOMA-B, homeostasis model assessment for beta cell function; HbA1c, hemoglobin A_1c_; TG, triglyceride; TC, total cholesterol; HDL-C, high-density lipoprotein-cholesterol; LDL-C, low-density lipoprotein-cholesterol

Data are given as mean ± standard deviation. *P* values represent statistical difference between different genotypes assessed by independent-samples t-tests. ^b^*P* values are determined by Pearson chi-square test. ^c^*P* values are determined by Kruskal–Wallis test

DV, differential values (postadministration minus preadministration)

### Association of PPARD rs2016520 and NOS1AP rs12742393 genetic polymorphisms with response rate to nateglinide treatment

In order to evaluate the association of *PPARD* and *NOS1AP* polymorphisms with the response to nateglinide treatment, genotypes and allelic frequency distributions were analyzed in the responder and non-responder groups (Table 4). No signifficant effects of the variation in *PPARD* rs2016520 (T/C) on nateglinide treatment were detected. According to predetermined criteria of 20% reduction from baseline, *NOS1AP* rs12742393 A allele carriers exhibited higher response rate to nateglinide treatment; AA allele homozygotes had the highest response rate (70.83%), while AC heterozygous and CC homozygous had 44.44% and 22.22%, respectively (*P* = 0.006).

**Table 4:** Genotype and allele distributions between responders and non-responders of *PPARD* rs2016520 and *NOS1AP* rs12742393 variants (n = 60)

*PPARD* rs2016520	Genotype			*P* value	Allele frequency		*P* value
	TT	TC	CC		T	C	
Responder (%)	19 (54.29%)	11 (47.83%)	1 (50.00%)		49 (52.69%)	13 (48.15%)	
Non-responder (%)	16 (45.71%)	12 (52.17%)	1 (50.00%)	0.964	44 (47.31%)	14 (51.85%)	0.678
*NOS1AP* rs12742393	AA	AC	CC		A	C	
Responder (%)	17 (70.83%)	12 (44.44%)	2 (22.22%)		46 (61.33%)	16 (35.56%)	
Non-responder (%)	7 (29.17%)	1 5 (55.56%)	7 (77.78%)	0.027	29 (38.67%)	29 (64.44%)	0.006

## Discussion

In the present study, we found for the first time that genetic polymorphisms of *PPARD* and *NOS1AP* may affect the therapeutic efficacy of nateglinide in Chinese patients with T2DM. We observed that, T2DM patients with at least one C allele of *PPARD* rs2016520 (T/C) or one C allele of *NOS1AP* rs12742393 (A/C) may be less responsive to treatment with nateglinide, indicating that the *PPARD* and *NOS1AP* genotype may serve as nateglinide response prognosticator. Therefore, we suggest that prior genotyping and individualized administration of nateglinide may be beneficial for those T2DM patients who require treatment with nateglinide. *PPARD* and *NOS1AP* are directly or indirectly involved in the regulation of β-cell function and insulin resistance, which suggests that genetic polymorphisms in the two genes may contribute to interindividual differences in nateglinide response [7–9, 16, 17]. Further pharmacogenetic and functional studies are necessary to investigate the potential mechanism and lay the foundation for Individualized administration for patients with T2DM.

Studies have reported that *CYP2C9* and *SLCO1B1* gene polymorphisms could affect the pharmacokinetic process of nateglinide, resulting in differences in drug concentrations in plasma and therapeutic efficacy [27–30]. Therefore, we selected patients with the same CYP2C9*1 and *SLCO1B1* 521TT genotype to avoid any possible changes in the pharmacokinetics and pharmacodynamics of nateglinide caused by *OATP1B1* or *CYP2C9* polymorphism. After the treatment with nateglinide for 8 consecutive weeks, serum FPG, PPG, HbA1c, TG and TC levels decreased significantly in patients with T2DM and FINS, PINS, HOMA-B and HDL-C levels increased markedly increased markedly. Also, patients with *PPARD* rs2016520 TC + CC genotypes had attenuated efficacy of nateglinide monotherapy with respect to PPG and HOMA-B compared with TT genotype carriers. Our results also showed that the *NOS1AP* rs12742393 (A/C) polymorphism was associated with an attenuated nateglinide effect in Chinese patients with T2DM, and that individuals with AC + CC genotypes showed a smaller increase in FINS and HOMA-B, but a smaller decrease in FPG and HOMA-IR levels as compared to individuals with the TT genotype.

*PPARD* encoding PPAR-δ, which is related to islet function and insulin resistance, might directly or indirectly participate in the pathogenesis of T2DM [9, 31–33]. In the present study, we preliminarily found that the polymorphism of *PPARD* affected the impact of nateglinide on insulin secretion in Chinese patients with T2DM, which may be attributed entirely to the role of PPAR-δ in insulin secretion, as measured by HOMA-B. The biological effect of PPAR-δ overlaps with the therapeutic mechanism of nateglinide to a certain extent, which can partly explain the mechanism of *PPARD* genetic polymorphism affecting the efficacy of nateglinide. However, the exact molecular mechanism remains to be further studied.

*NOS1AP* mainly regulates nNOS activity, and nNOS can inhibit intracellular Ca^2+^ level and thereby regulate insulin secretion [15, 18, 34]. In addition, lateral ventricular injection of nNOS inhibitors can affect insulin secretion and insulin sensitivity [15]. In this study, we observed that subjects with at least one C allele of the *NOS1AP* rs12742393 showed a smaller decrease in FPG and HOMA-IR and more obvious increase in FINS and HOMA-B levels than those with the AA genotype, which suggested that the *NOS1AP* rs12742393 C allele confers the poor nateglinide response through improving insulin resistance, as measured by HOMA-IR. Animal studies have shown that knockout of mouse nNOS gene may induce insulin resistance in mice [35]. Meanwhile, it has been reported that the dysfunction of nNOS in islet β cells is related to insulin secretion [15, 16]. Therefore, it is speculated that *NOS1AP* rs12742393 risk gene C affects the efficacy of nateglinide in patients with T2DM, which is at least partially associated with insulin resistance and islet β cell. However, the exact mechanism by which *NOS1AP* polymorphism affects the efficacy of nateglinide needs to be further investigated.

In interpreting the results of our study, several shortcomings must be addressed. First, our study focused only on the effect of variation in *PPARD* rs2016520 and *NOS1AP* rs12742393 on nateglinide efficacy. However, the possibility still exists that other susceptibility loci for T2DM may affect the therapeutic efficacy of nateglinide. Second, the sample size was relatively small, we may have missed some meaningful results. The duration of nateglinide therapy for 8 weeks is relatively short, since HbA1c reduction is better observed after 12 weeks of treatment. Further studies with a larger sample size and longer observation periods are required to confirm the effects of *PPARD* and *NOS1AP* polymorphisms on the therapeutic efficacy of nateglinide. Third, our study only investigated the effects of gene polymorphism on the efficacy of nateglinide. However, the mechanisms by which the two SNPs in *PPARD* and *NOS1AP* affect the therapeutic efficacy of nateglinide are not fully understood. In the future, more functional studies are needed to explore the mechanism by which *PPARD* and *NOS1AP* genetic polymorphisms affect drug efficacy.

## Conclusion

The efficacy profile of primary diabetic patients receiving nateglinide monotherapy was associated with *PPARD* rs2016520 and *NOS1AP* rs12742393 polymorphisms. Prior genetic analysis of *PPARD* rs2016520 (T/C) and *NOS1AP* rs12742393 (A/C) is a useful idea worthy of further exploration to achieve individualized drug delivery.

## Data Availability

The datasets generated and analyzed during the current study are available in the link “https://submit.ncbi.nlm.nih.gov/subs/variation_file/SUB9598166/overview”. The accession number is SUB9598166.

## Abbreviations

T2DM: Type 2 diabetes mellitus
FPG: Fasting plasma glucose
PPG: Post plasma glucose
BMI: Body mass index
PCR–RFLP: Polymerase chain reaction-restriction fragment length polymorphism
TG: Triglycerides
TC: Total cholesterol
LDL-c: Low-density cholesterol
HDL-c: High-density cholesterol
HbA1c: Glycated hemoglobin
HOMA-IR: Homeostasis model assessment for insulin resistance
HOMA-β: Homeostasis model assessment for islet β cell function
FINS: Fasting serum insulin
PINS: Postprandial serum insulin
